# Making the message clear: visualizing mRNA localization

**DOI:** 10.1016/j.tcb.2010.03.006

**Published:** 2010-07

**Authors:** Timothy T. Weil, Richard M. Parton, Ilan Davis

**Affiliations:** Department of Biochemistry, University of Oxford, South Parks Road, Oxford OX1 3QU, UK

## Abstract

Localized mRNA provides spatial and temporal protein expression essential to cell development and physiology. To explore the mechanisms involved, considerable effort has been spent in establishing new and improved methods for visualizing mRNA. Here, we discuss how these techniques have extended our understanding of intracellular mRNA localization in a variety of organisms. In addition to increased ease and specificity of detection in fixed tissue, *in situ* hybridization methods now enable examination of mRNA distribution at the ultrastructural level with electron microscopy. Most significantly, methods for following the movement of mRNA in living cells are now in widespread use. These include the introduction of labeled transcripts by microinjection, hybridization based methods using labeled antisense probes and complementary transgenic methods for tagging endogenous mRNAs using bacteriophage components. These technical innovations are now being coupled with super-resolution light microscopy methods and promise to revolutionize our understanding of the dynamics and complexity of the molecular mechanism of mRNA localization.

## Introduction

The question of when and where genes are expressed has been of major interest in biology for at least 50 years. Although the study of the spatial positioning of transcripts initially focused on differences in expression levels between tissues, approximately 30 years ago it was realized that transcripts can also localize asymmetrically within cells. Intracellular localization of mRNA is now thought to be a very common mechanism to target protein function, occurring in most eukaryotic model organisms and for a very wide range of transcripts in the genome. mRNA starts its life as nascent transcripts that are first processed and then exported from the nucleus into the cytoplasm. Such transcripts associate in the nucleus with RNA binding proteins to form ribonucleoprotein complexes (RNPs), whose composition is then thought to be extensively remodeled during export from the nucleus and over the subsequent life cycle of the mRNAs in the cytoplasm. Specific RNA binding proteins within RNPs play essential roles in mRNA localization, translational regulation and degradation [1–3]. Since the 1980s, when the link between mRNA localization and protein targeting was established, there has been considerable interest in intracellular imaging of the distribution of mRNAs [4,5]. Initially, only *in situ* hybridization (ISH) on fixed samples was available to study intracellular mRNA localization. More recently, technical advances have allowed the visualization and quantitation of mRNA movement in living cells, enabling more effective analysis of the molecular mechanisms involved. In this review, we discuss key advances in mRNA labeling and detection approaches, imaging instrumentation, post acquisition analysis and the impact this has made on the field.

## In the beginning there was ISH

**Figure 1:**
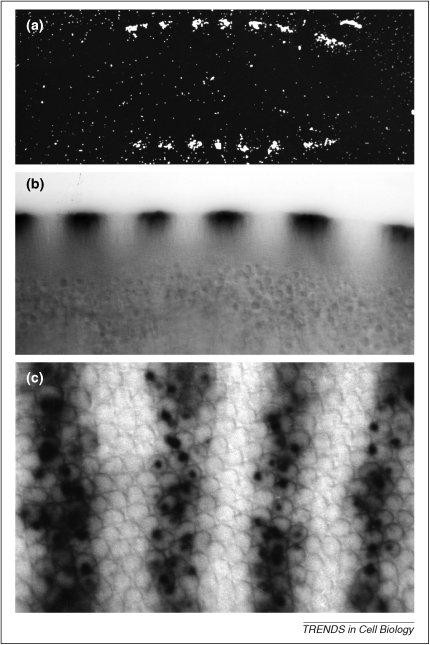
Detecting RNA in fixed cells. **(a)** ISH on 5-μm-thick wax sections of *Drosophila* syncytial blastoderm embryos using a tritiated probe against *fushi tarazu* pair-rule transcripts that are expressed in seven stripes. Silver grains of the photographic emulsion were visualized using dark field microscopy. **(b)** ISH on a whole-mount *Drosophila* syncytial blastoderm using a DIG-labeled probe against transcripts encoding the Even-skipped pair-rule protein detected by an anti-DIG alkaline-phosphatase-coupled antibody highlighted by histochemical staining using NBT and X-phosphate. The dark histochemical precipitate was imaged in bright field and photographed with black-and-white photographic film. **(c)***Drosophila* syncytial blastoderm treated as in **(b)**. The shallow view shows expression stripes of nascent transcripts within nuclei. (Taken from I. Davis D.Phil. Thesis.)

When intracellular mRNA distribution was being established as a mechanism for creating embryonic asymmetry [6], ISH in fixed samples was the only available method for examining the distribution of transcripts in fixed samples. Radioactively labeled probes were detected in wax tissue sections as silver grains in a photographic emulsion that coated the section [7]. This method of detecting a signal through the accumulation of silver grains (Figure 1a), although having the advantage of being quantitative, required a high degree of skill and considerable patience, because a typical exposure time was approximately 1 month. Therefore, it was a major advance when a histochemical detection method became available using alkaline-phosphatase-coupled antibodies that detect Digoxigenin (DIG) labeled probes (Figure 1b,c) [8]. This provided a 2-day procedure that, although not as quantitative as counting silver grains, was highly sensitive and demanded considerably less skill from the researcher.

The advent of fluorescent methods for detecting transcripts enabled higher quality three-dimensional imaging, multiplexing different RNA species and co-visualization of RNA with proteins [9]. For example, the distribution of *trans*-acting factors could be investigated with a high degree of accuracy while co-visualizing the RNA [4]. Fluorescent detection of RNA was first achieved using fluorochrome labeled antibodies that detected DIG or biotin incorporated into the probes [10] and later by a histochemical staining procedure known as tyramide signal amplification [11]. However, histochemical staining methods are only semi-quantitative, because the signal varies between experiments or batches of reagents and is non-linear by nature [12]. A modified version of this protocol, allowing deeper penetration of probes, has been used to characterize the localization of 3000 transcripts in the *Drosophila* blastoderm embryo [13]. Of these, a majority show a distinct intracellular localization as opposed to uniform distribution.

**Figure 2:**
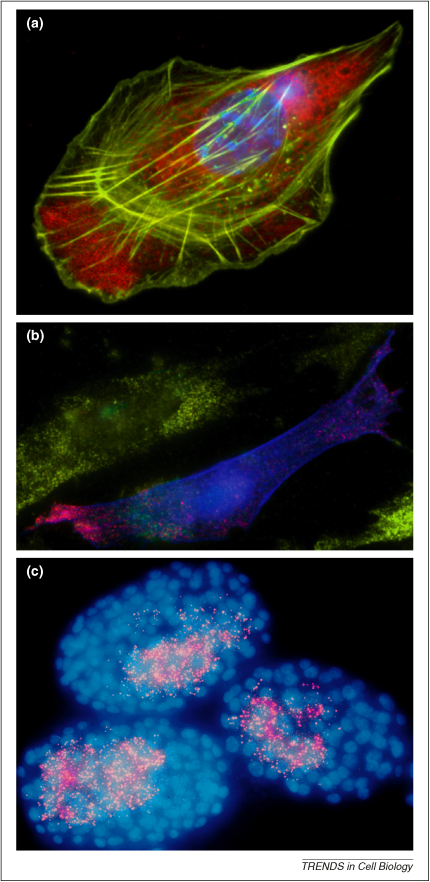
Detecting RNA in fixed cells by fluorescence. **(a)** Indirect labeling of RNA through its association with ZBP1. Double indirect immuno-fluorescence of a fixed primary mouse embryo fibroblast shows that ZBP1 (mouse CRD-BP), pseudo-colored in red, localizes to the leading edge of a migrating cell. The actin cytoskeleton (green) is detected using a pan-actin antibody followed by an Alexa 488 labeled secondary antibody. Note that CRD-BP is excluded from the lamellipodia. The nucleus is stained with DAPI (blue) (courtesy of the Singer Lab). **(b)** Multiple fluorescence in situ hybridization (FISH) to compare the distribution of two RNA species. Primary chick embryo fibroblast cells were transfected with an eGFP-β-actin DNA plasmid containing the full-length human β-actin 3′ UTR followed by 24 MCP binding sites. The middle cell expresses human β-actin protein (blue) following successful transfection. The exogenous RNA (red) was detected specifically by FISH using probes against the *MS2* stem-loops. Endogenous β-actin mRNA (green) was also detected using FISH (courtesy of the Singer Lab). **(c)** Single molecule FISH in *C. elegans* N2 embryos showing individual *elt-2* mRNAs (red dots). The nuclei are highlighted by DAPI staining of DNA (blue) (courtesy of Arjun Raj) [17].

ISH has been used in a variety of different organisms and cell types with varying success. In tissue culture cells, for example, the technique is particularly successful (Figure 2a,b) [14,15]. In neuronal tissues, such as the neuromuscular junction, the technique has proven harder to implement [16]. The problem in this tissue appears to be a combination of penetration and amplification. In *Caenorhabditis elegans,* improvements in direct labeling techniques have recently solved the long standing difficulties with ISH in this tissue, allowing the successful mapping of RNA distributions (Figure 2c) [17]. Single RNA molecules have been reported by quantitative fluorescent detection [17,18] and through the systematic quantification of multiple oligo probes with fluorescently labeled nucleotides at predefined positions [15,19]. The intensity of individual oligos can be measured *in vitro* and then related to the intensity of signal detected on a fixed specimen to determine the number of RNA molecules represented by a given fluorescent signal.

ISH techniques have also been extended to include ultrastructural analysis. One method of detection is to inject a gold particle coupled RNA into cells, allow localization, then fix and image with a transmission electron microscope (TEM) [20]. A more generally applicable EM-level ISH technique involves hybridization of antisense probes on ultrathin sections (60–100 nm) and subsequent detection with gold-coupled antibodies [21–24] (Box 1a,b). This technique provides excellent high-resolution detection of mRNA coupled with ultrastructural information (Box 1c–e). However, an EM approach faces limitations that include intricate sample preparation, susceptibility to artifacts and low labeling efficiency. In the future, increasing the sensitivity of detection would be an important technical advance.

Today, ISH is still a commonly used technique for examining the cellular distribution of mRNA in fixed tissue. However, to further explore mRNA transport dynamics, maintenance mechanisms and localization machinery, observation of mRNA in live tissue is required.

## Live-cell imaging of mRNA

One of the biggest challenges to performing live cell imaging is how to label the RNA in the living cell. Some of the first attempts to image RNA in living cells relied on feeding cells fluorescent labeled metabolites that are subsequently incorporated into transcripts [25]. However, this method is unsuitable for the detection of specific transcripts. Labeling individual species of transcripts can be achieved by first incorporating amino-allyl nucleotides during synthesis of RNA *in vitro* and subsequent chemical coupling to fluorescent derivatives. The resulting fluorescently labeled RNA can be introduced into living cells by microinjection. In *Xenopus*, injections into oocytes have been a key assay for elucidating the mechanism of Vg1 mRNA localization [26,27]. Similarly, in *Drosophila*, RNA labeled in this way was used to show that *oskar* (*osk*) mRNA can at least partly localize if injected near its final destination at the posterior of the oocyte [28] and in the embryo, microinjection of pair-rule transcripts has been used to study the mechanism of localization [29].

**Figure 3:**
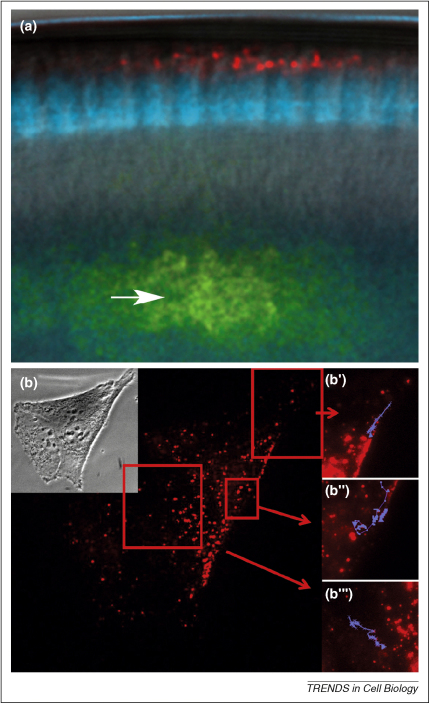
Detection of RNA in live tissue. **(a)** Localization of multiple RNAs via injection. *Drosophila* syncytial blastoderm expressing nuclear GFP (blue) first injected with an Alexa Fluor 546-labeled *runt* RNA (red) and shortly after with an Alexa Fluor 488-labeled *runt* RNA (green). Both RNAs were injected into the same site (arrow). The image shows the first RNA is already apically localized, whereas the second is in transit (courtesy of Renald Delanoue). **(b)** Tracking individual β-actin mRNA molecules *in vivo* with MTRIPs in a human epithelial cell. Images were taken at 0.5 Hz for 5 min. Areas of interest (b′–b′″) show trajectories of single mRNA granules (courtesy of Philip Santangelo) [34].

More recently, amino-allyl labeling has been replaced by direct incorporation of Alexa Fluor-labeled nucleotides into the transcript *in vitro*. Injecting RNA labeled in this way into live cells led to the formation of bright RNP particles (Figure 3a), and analysis of their movements revealed that transport occurs along microtubules by the molecular motor dynein and enabled one of the earliest determinations of RNA transport particle speeds [30]. This assay has also facilitated the characterization of *cis*-acting elements within the RNA sequence and the requirement for *trans*-acting factors [31,32]. Whereas *in vitro* RNA synthesis is very rapid and does not require lengthy transgenic experiments, the microinjection procedure is potentially damaging, and introducing a large excess of RNA can saturate intracellular machinery leading to inappropriate localization and expression.

ISH methods to detect mRNA in fixed material relies upon ‘melting’ and ‘annealing’ steps to open the target RNA sequence and permit specific binding of the RNA probe. However, methods based upon the hybridization of a labeled linear RNA probe have been extended to live imaging applications by using RNA variants such as 2′-O-methyl-RNA, that permit selective binding at 37 °C [33]. The most recent implementation of this type of approach is the multiply labeled tetravalent RNA imaging probes (MTRIPs) method [34], which allowed detection of individual molecules of native β-actin mRNA in a human epithelial cell line (Figure 3b).

A related approach to achieve specific labeling of RNA using introduced nucleic acid based probes is through the introduction of ‘molecular beacons’ into cells [35,36]. The beacons technique makes use of an RNA probe in the form of a small hairpin loop labeled at the free ends with a fluorescent dye and a molecule that quenches the fluorescence emission of that dye, respectively. When unbound to an endogenous RNA, the probe adopts a looped conformation so that it folds back on itself bringing the quencher into close proximity with the dye, thus preventing fluorescence emission. By contrast, when the RNA probe is bound to the target sequence, it unloops and the quencher is kept away from the fluorochrome leading to fluorescence emission. In this way, beacons are expected to improve contrast over fluorescently labeled probes, which generate substantial background fluorescence when unbound to their target sequence. Additionally, a modification of this technique is to use fluorescence resonance energy transfer (FRET) to improve probe specificity [33]. The idea is to use a pair of beacon probes that bind to adjacent sequences on a single transcript. The relative positions of the fluorochromes and quencher molecules are such that when bound to the target sequences, the two fluorochromes lie adjacent within FRET distance. Selectivity is gained by the necessity of binding two probes. Using cell permeabilization [37], ‘cell-penetrating peptide’ conjugation (such as TAT-1) [38] or injection to introduce the probes [35], this method has been successfully employed in multiple experimental systems to detect RNAs. Although very elegant in concept, the technique has not yet been as widely utilized as other methods. In *Drosophila*, molecular beacons were used to follow *osk* RNA, an abundant posteriorly localizing transcript [35]. However, beacons have not yet been successfully used to follow other less abundant RNAs in *Drosophila*. This might be owing to a limitation in the maximal possible brightness when using one fluorochrome per RNA target, so brighter beacons or multiple beacons per target could overcome this problem in the future.

An ideal approach for following transcripts in living cells, would be to label endogenous mRNAs very brightly and follow their native and complete pathway for synthesis, movement, localization and degradation. Such a method would be achieved without physical disruption to the specimen. Bacteriophage RNA binding proteins certainly have some of these properties, and have become very popular for labeling RNA *in vivo*. However, as we will see, they too have some drawbacks, and for this reason direct introduction of fluorescently labeled RNA or hybridization probes is still being used as a complementary approach.

## *MS2*-MCP system

Imaging of mRNA *in vivo* was revolutionized by the advent of the *MS2*–MCP system (Box 2a), which enables mRNA expressed in transgenic animals to be decorated *in vivo* with fluorescent proteins (FPs) (Box 2b–e) [39]. The *MS2* system was pioneered by Singer and co-workers in yeast to track the movement of *ASH1* mRNA into the daughter cell or bud during cell division [39,40]. Two components are necessary for labeling: (i) expression of the mRNA of interest, modified to include the *MS2* stem–loop motifs; (ii) the *MS2* RNA coat protein (MCP) fused to an FP expressed either globally or in chosen tissue. With the *MS2* system*,* the fusion protein has been shown to bind to the RNA motif in the modified mRNA with a dissociation constant of 5 nM, which is essentially irreversible in the lifetime of an experiment. This means that, once associated, the components are effectively permanently coupled [41]. When the system is used with endogenous promoters to drive expression of the *MS2*-tagged mRNA, physiological levels of mRNA are produced that follow the complete mRNA localization pathway.

The same technology has been applied to other systems with great success, facilitating the analysis of the mechanism of mRNA localization in a variety of organisms [42–49]. *MS2* tagging has proven to be particularly valuable in situations where ISH or injection of labeled RNA has proven impractical [42,50] (for a detailed table, see Ref. [51]). In *Drosophila*, the *MS2* system has been indispensable for studying mRNA localization in late oogenesis when ISH is unreliable owing to the follicle cells laying down the chorion and vitelline membrane [43,52] and in early oogenesis when microinjection is not very effective [48].

Although the *MS2-*MCP system has been used widely, the drawbacks of this method include the necessity of making transgenic animals that express the constructs and the fact that there are no clear rules for choosing where best to place RNA stem–loops in the endogenous transcript. Another difficulty that can arise is that the MCP-FP fusion itself can become localized in the cytoplasm in a tissue dependent manner or aggregate into particles without the RNA being expressed. There is also a limit on the number of loops that can be introduced into the endogenous mRNA because of the difficulty in cloning repeats and the effect of increasing the size of the RNA considerably. This limits the brightness of the MCP tagged mRNA. Another difficulty that can arise is nonspecific fluorescence in the cytoplasm or the formation of fluorescent aggregates in tissue where the MCP-FP fusion is expressed without the RNA. Despite these drawbacks, *MS2*–MCP has become an invaluable method for following transcripts *in vivo*.

## Optimizing the imaging of labeled RNA *in vivo*

Labeling RNA is only the first challenge to be met when studying RNA localization, because it is also crucial to consider cell viability, the most appropriate imaging approach and the use of post acquisition analysis. Quantitative live cell imaging enables the detection of subtle differences in movement and phenotypes, which is particularly powerful when used in combination with transport inhibitors and in mutant backgrounds.

### Choosing an imaging approach

Matching the experimental approach to the imaging method is essential for all live-cell imaging situations [53]. The speed of imaging, resolution, sensitivity and the effects on cell viability all need to be taken into account. RNA localization typically involves small, weakly labeled and rapidly moving transport particles. Frequently, larger static or slow-moving particles are not the major element of transport. To detect all aspects of localization, different microscopic approaches are available. Wide-field microscopy with deconvolution using CCD detectors has both the speed and sensitivity to follow the dynamics of mRNA localization [49,54,55]. Although less sensitive in detecting photons than wide-field, spinning disc confocal imaging gives increased contrast in thicker specimens or specimens that have a higher background signal [56]. Laser scanning confocal imaging is better at removing out-of-focus light than a spinning disc system but is generally not appropriate for following highly dynamic processes owing to a lack of speed and sensitivity. It is often desirable to co-visualize the movements of RNAs with those of proteins such as *trans*-acting factors or cytoskeletal components. Multi-channel imaging of rapidly moving objects presents some considerable technical challenges. Most obvious is the requirement to minimize crosstalk between detection channels. To resolve the need to maximize photon collection while imaging multiple channels simultaneously, multi-detector systems coupled with spectrally well-separated probes and appropriate filter sets are utilized. The optical microscope experimental (OMX) system, with fast live multichannel imaging, designed in the Sedat lab, is one such multi-detector system [57,58].

### Analysis and quantification

Tracking sub-micron mRNA transport particles moving at speeds on the order of 1–3 μm/s requires objective and precise data analysis. Image analysis can be separated into three aspects: (i) pre-processing to improve the quality of data – this includes deconvolving, denoising and contrasting images [59,60]; (ii) segmentation to identify objects of interest (one simple approach is intensity-based thresholding for specific object recognition); (iii) counting, measuring intensity or tracking the movement of the objects of interest. Many commercial packages exist with automated or semi-automated analysis and tracking tools. Generally, these only work with images that have a high signal-to-noise ratio and high contrast. Custom analysis strategies, for example written in MATLAB, extend the range and power of automated tracking and other image analysis approaches [61].

## The future is bright

**Figure 4:**
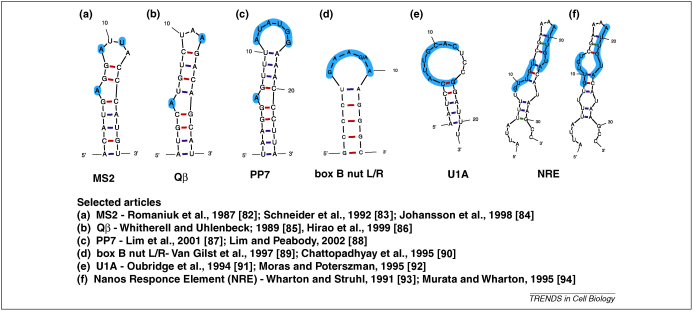
Different options for endogenous tagging of mRNA*s in vivo*. UNAfold (by Stewart and Zuker (http://dinamelt.bioinfo.rpi.edu/download.php) predicted secondary structures for RNA stem–loop sequences which could be inserted into RNA constructs for labeling mRNA. Highlighted regions indicate nucleotides important for the binding specificity of the corresponding binding protein [82–94].

Powerful as it is, the standard *MS2*–MCP based method is not sufficient to address all questions, in all circumstances. For example, it is often necessary to follow several mRNA species simultaneously, image mRNA movement within thick specimens or resolve images where considerable autofluorescence is present. One emerging approach is the development of alternative labeling systems (Figure 4) that could be used in parallel to the *MS2*–MCP system to enable multiple mRNAs to be imaged simultaneously. An example is the use of the lambda boxB RNA sequence (Figure 4d) binding to the peptide N, another is the U1A RNA (Figure 4e) and its binding protein. A complementary approach has been to use an engineered version of the human form of Pumilio (hPum) targeting specific RNA sequences (Figure 4f). The structure of this interaction is well characterized, as are the rules for modifying the amino acid residues required to bind to a specific short RNA sequence. One elegant application of this technique has been through the use of split GFP protein fusions linked to Pum proteins, eliminating unwanted background fluorescence from the unbound FPs, with the possibility that a small amount of fluorescence could remain after the RNA is degraded [62,63]. A signal is observed when two split GFP fusion proteins are both bound to adjacent Pum-binding sites on a RNA, thus allowing the two halves of the split GFP to come together and become fluorescent. However, the possibility remains that a small amount of fluorescence persists after the RNA is degraded. Another exciting imaging advance is through the use of new far-red-emitting FPs as well as photo-switchable and photo-activatable constructs [64–66]. Owing to the longer wavelength of excitation, far-red facilitates clearer imaging in thick specimens, whereas photo-convertible proteins allow for the imaging of a spatially restricted population of RNA molecules with much better contrast and will be a powerful alternative to fluorescence recovery after photobleaching (FRAP), for studying RNA anchoring.

In all cases, the success of a fluorescent assay can be greatly enhanced by advances in detection and analysis methods. Most recently, there has been considerable activity in developing new light microscopy methods that exceed the resolution limits of conventional optical microscopy of 200 nm [67]. Key developments in this area have included super-resolution imaging methods such as photo-activation light microscopy (PALM), stimulated emission depletion (STED) and three-dimensional-structured illumination microscopy (3D-SIM).

### PALM and related techniques

This technique makes use of the fact that imaging a single point source of fluorescence produces a diffraction-limited image spot from which an exact central position (centroid) can be determined at precisions that greatly exceed the resolution limits of light microscopy [68,69]. In complex samples, this technique works by iteratively building up an image from the emissions of individual dye molecules. To achieve this, certain dyes are used that can be stochastically photo-activated (as in PALM) or photo-switched [as in stochastic optical reconstruction microscopy (STORM)], a small number of molecules at a time. These single-molecule point sources are imaged, then the sequence of activating and imaging is repeated with different sets of molecules to build up an overall image. In this way, an image is built up with a precision of better than 20 nm for each molecule. Although conventional optics can be used with the appropriate activation/collection imaging regime, this restricts the technique to samples that are very thin, such as fixed sectioned material. Alternatively, total internal reflection microscopy (TIRFm) illumination can be used to increase axial resolution to approximately 100 nm, although this restricts imaging depth to within 200 nm of the coverslip, imposed by the TIRFm method itself [68]. Although the low photon flux and iterative imaging regime of PALM make the application of the technique to living cells difficult, this has been achieved in specific cases [70].

### STED

This is a true optical technique that works by ‘sculpturing’ the emission of light from a laser-scanned specimen [67]. In this case, the ability to prevent dye fluorescence by irradiation with high levels of a different wavelength of light is exploited. The emission from an irradiated spot is effectively constrained in the X–Y axis to well below the diffraction limit of the light. The high irradiation energies required to collect images and the restricted number of dyes that can be used has meant that live cell imaging has only been possible in particular cases involving very bright specimens [71–73]. When combined with 4Pi [74], super-resolution can be achieved in the X–Y and Z planes. However, the combination of STED and 4Pi puts considerable constraints on imaging, especially in terms of the complexity of the equipment required and sample preparation.

### 3D-SIM

Currently implemented on the OMX system (Box 3) [58], this technique allows doubling of the resolution in the X–Y and Z planes (Box 3c–g). This technique can be utilized with conventionally fixed and prepared multi-labeled samples [75]. 3D-SIM uses a ‘structured illumination’ (SI) excitation regime in which 15 striped patterns of light are sequentially imposed on the specimen in each imaging plane. These patterns interact with features of the specimen to generate patterns in the resulting images. Post-acquisition image processing of the multiple SI images extracts the super-solution features. On OMX, the technique is currently too slow to use on live material, but alternative SI implementations using TIRF to achieve greater Z resolution have been used for live-cell super-resolution imaging at intervals of 100 ms per frame [76]. To date, these techniques have had limited application to questions of RNA localization [57]. However, we believe that they hold considerable promise in exploring the molecular components of RNA transport particles, cellular machinery of anchoring and the regulation of translational control. One technical barrier that will need to be overcome in the future is increasing the speed of 3D-SIM to allow live cell imaging at the increased resolution.

## Imaging faster and for longer

One of the obstacles to studying RNA localization is being able to follow the fate of localizing transcripts from transcription through translation to degradation. A major limiting factor with long term imaging at high spatial and temporal resolution is dye bleaching. Recent developments in improved sensitivity of imaging, denoising algorithms and dyes with improved brightness and stability hold the promise that long term RNA imaging, to follow the whole lifecycle of the RNA, is achievable.

### Quantum dots

Fluorescence nanoparticles, quantum dots, are now commercially available and have successfully been used for long term imaging of single molecules in cells [77,78]. Quantum dots are fluorescent micro-crystals that are extremely bleach resistant, have a narrow excitation spectrum in the blue region (404–440 nm) and can be selected by size to give a range of emission colors with very narrow bandwidths. Together, these characteristics make them ideal for long term and multiplexed labeling. Quantum dots have been successfully used in multiplex *in situ* [79,80] and are currently being applied to live-cell imaging of RNA [81]. Despite their promise, there have been some major obstacles to using quantum dots. They exhibit ‘blinking’ where their ability to fluorescence turns off and on, which is a problem when tracking a single or sparsely labeled molecule. They can bind nonspecifically in tissue, but efforts are being made to improve ‘biological compatibility’ via coatings of the dots. The relatively large size, compared with conventional dyes, leads to cell penetration problems, steric hindrance difficulties when associated with target molecules and the presence of multiple ligand interaction sites on the surface of each quantum dot. Multiple interacting sites can cause ‘blocking’ of target sites resulting in reduced detection sensitivity or crosslinking of molecules when labeling RNAs. Nevertheless, it is possible to isolate derivatized particles with a particular number of ligands bound to the surface and researchers are working on nanoparticles with a single ligand binding site.

### Post-acquisition image processing

These methods have also been making a considerable contribution, for example, ‘denoising’, to improve the quality of images acquired with very low excitation times [59]. By allowing useful image data to be obtained with very few photons, denoising helps to avoid photo-damage and photo-bleaching, which is especially important when imaging a time-series in live tissue. Improvements in automated particle tracking algorithms will also make an invaluable contribution although improved quantitative analysis of live-cell data [61]. Many of these techniques are only just being applied to RNA visualization, but it is clear that they will have a major impact in the future.

## Concluding remarks

mRNA localization remains an intense area of investigation and with the tools being developed the field is poised to tackle long standing and new questions. The ongoing development of bacteriophage based tagging systems will allow the simultaneous imaging of multiple RNAs together with proteins and cytoskeletal components in living cells. Improvements in image processing and RNA tracking methods coupled with new generation of fast simultaneous three-dimensional multi-color imaging systems will allow a quantitative evaluation of the entire path of movement of transcripts in cell. Single particle live imaging in synergy with super-resolution methods and electron microscopy will allow researchers to elucidate the changes in RNP composition that occur during the life cycle of single RNA molecules. We await the application of these new methods with great interest.

## Figures and Tables

**Figure I:**
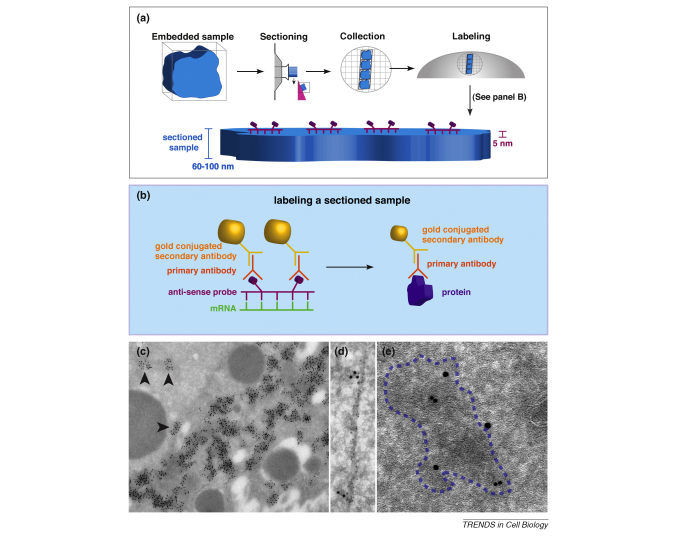
ISH-IEM on ultrathin frozen sections.

**Figure II:**
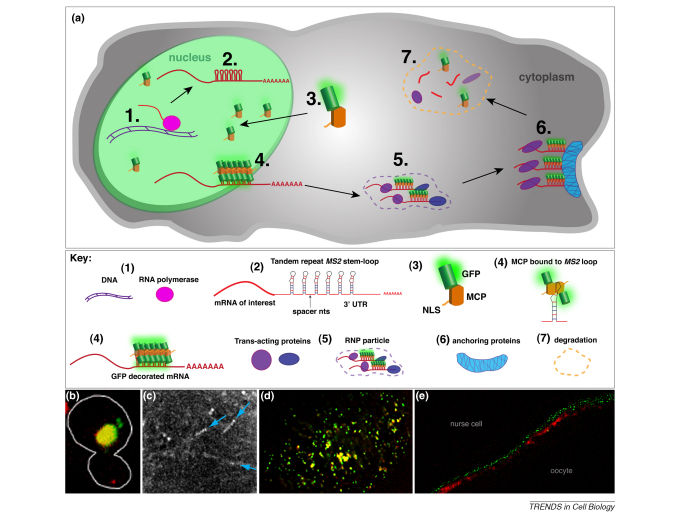
*MS2*-MCP labeling the life cycle of endogenous mRNA.

**Figure III:**
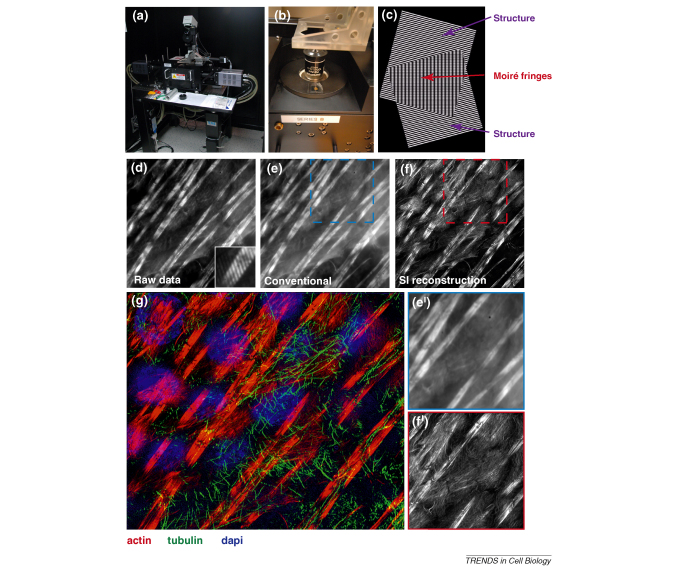
Super-resolution imaging by SI on the Sedat OMX.

## Box 1 In situ hybridization immuno-electron microscopy (ISH-IEM) on ultrathin frozen sections

(Adapted from methods described in detail in Delanoue et al., 2007 [22] and Herpers and Rabouille, 2004 [23].) (Figure Ia) Fixed samples are first embedded in gelatin and left overnight in sucrose to eliminate moisture from the sample. Samples are trimmed of excess gelatin and mounted on a pin for sectioning by cryomicrotome (at −120 °C). Multiple sections, each 60–100 nm thick, form a ribbon that is retrieved and placed on carbon-coated formvar nickel grids. Washing and labeling of the sectioned sample on the grids are carried out by floating the grids on drops of solution. (Figure Ib) ISH and labeling of sectioned samples. The DIG or biotin labeled RNA anti-sense probe is allowed to hybridize and then detected by first a primary antibody against the labeling epitope and then by a secondary antibody conjugated to gold. For a subsequent protein labeling, the sample is fixed and labeled with a primary antibody for the protein of interest and a secondary antibody conjugated to a different sized gold particle. (Figure Ic) ISH-IEM for *oskar (osk)* mRNA in a stage 9 wild-type *Drosophila* oocyte shows *osk* RNPs at the posterior pole area in large particles (arrowheads) and *osk* mRNA accumulation at the posterior pole, within electron-dense aggregates (Trucco et al., 2009 [24], adapted with permission). (Figure Id) ISH-IEM for *gurken (grk)* mRNA in a stage 9 wild-type *Drosophila* oocyte (15 nm) detected in transport particles associated with a microtubule (5 nm). These RNP particles are probably undergoing active transport toward the oocyte nucleus (Delanoue et al., 2007 [22], adapted with permission). (Figure Ie) IEM detecting an RNP particle of *bicoid (bcd)* mRNA. As an alternative to ISH, *bcd* mRNA tagged with GFP using the *MS2* system (bcd*GFP) in a stage 13 *Drosophila* egg chamber was detected using anti-GFP antibody (10 nm) followed by anti-Dynein heavy chain (15 nm) labeling. This shows bcd*GFP in a particle (dashed outline) together with dynein motor components (Weil et al., 2010 [57], adapted with permission).

## Box 2 MS2-MCP labeling the life cycle of endogenous mRNA

(Figure IIa) A *trans*-gene encoding the RNA of interest, with the *MS2* stem-loop sequences, is transcribed by RNA polymerase in the nucleus (1) resulting in endogenous mRNA of interest with tandem repeats of the *MS2* stem–loops (2). In most cases, constructs have included 6, 12 or 18 copies of the stem–loops, separated by random nucleotide (nts) sequence spacers, commonly introduced into the 3′ or 5′ UTR. (3) The MCP fusion to green fluorescent protein (GFP) is translated in the cytoplasm. Constructs expressing other FPs can be utilized for different tissues to avoid autofluorescence or increase levels of detection. Excess MCP-GFP is sequestered in the nucleus owing to a nuclear localization signal (NLS) engineered in the construct, this decreases fluorescent background in the cytoplasm when imaging. MCP-GFP binds as a dimer to each *MS2* stem–loop (4), decorating the mRNA of interest with FPs. Tagged mRNA form ribonucleoprotein (RNP) complexes with *trans*-acting factors (5). These particles include stabilizing, translational control and localization proteins. (6) mRNA is anchored at the cellular destination. Anchoring can involve components of the cytoskeleton, molecular motors and/or anchoring proteins. Following translation, localized mRNAs are degraded (7). Improvements in brightness and contrast are achieved by introducing additional tandem repeats of the *MS2* stem–loop into the mRNA construct; balancing expression levels of the *mRNA-(ms2)* and MCP-GFP; restricting expression of constructs to specific cell types using the UAS/Gal4 system or specific promoters. (Figure IIb) Simultaneous detection of multiple mRNA species in live yeast. *Saccharomyces cerevisiae* showing two differentially tagged mRNAs: *IST2-boxB* (red) and *HOM2–MS2* (green). Colocalization in yellow; cell border outlined (courtesy of the Jansen Lab). (Figure IIc) mRNA particles detected moving directly on microtubules. *bicoid (bcd)* mRNA tagged with GFP using the *MS2* system (bcd*GFP) and tau-GFP near the anterior cortex of a stage 12 *Drosophila* oocyte (Weil et al., 2008 [52], adapted with permission). (Figure IId) *bcd* mRNA particles colocalized with the double-stranded RNA binding protein Staufen (Stau) protein, which is required for *bcd* mRNA localization. The majority of bcd*RFP at the anterior of a stage 12 oocyte colocalizes with Stau (green) (Weil et al., 2010 [57], adapted with permission). (Figure IIe) Swallow (Swa) protein, required for *bcd* mRNA localization, co-visualized but not colocalizing with *bcd* mRNA. bcd*RFP and Swa-GFP are detected at the anterior cortex of a stage 12 oocyte using the SI super-resolution mode of OMX (see Box 3). At this resolution, Swa protein and *bcd* mRNA have a distinct spatial distribution without overlap, suggesting that unlike Stau, Swa is not directly involved in *bcd* mRNA localization (Weil et al., 2010 [57], adapted with permission).

## Box 3 Doubling the resolution for light microscopy

Super-resolution imaging by SI on the Sedat OMX. (Figure IIIa) The custom built OMX microscope body, photographed inside its environmentally controlled chamber. (Figure IIIb) OMX stage and objective mounting designed specifically for exceptional thermal and mechanical stability. (Figure IIIc) The principle behind SI: the specimen is illuminated by a pattern of stripes, generated by a diffraction grating in the excitation path. This ‘structured’ pattern interacts with sub-resolution structures in the specimen. Distortions in the imposed pattern are imaged and the sub-resolution information ‘extracted’ by post acquisition processing. The effect is similar to that of Moiré fringes observed when two patterns interact to produce very obvious larger scale new pattern. (Figure IIId) Unprocessed SIM image of actin cables collected on OMX. The ‘structured’ pattern is visible as stripes within the image (inset). (Figure IIIe) Conventional wide-field image for comparison. (Figure IIIe′) Region of interest shown at increased magnification. (Figure IIIf) SIM reconstruction exhibiting increased resolution. (Figure IIIf′′) Region of interest shown at increased magnification. (Figure IIIg) SIM reconstruction of three channels showing the spatial relation between microtubules (green), actin bundles (red) and nuclei (blue) in a *Drosophila* egg chamber.
